# The effectiveness bundling of zinc with Oral Rehydration Salts (ORS) for improving adherence to acute watery diarrhea treatment in Ethiopia: cluster randomised controlled trial

**DOI:** 10.1186/s12889-016-3126-6

**Published:** 2016-05-31

**Authors:** Samson Gebremedhin, Girma Mamo, Henock Gezahign, Jacqueline Kung’u, Abdulaziz Adish

**Affiliations:** School of Public and Environmental Health, Hawassa University, Hawassa, Ethiopia; Micronutrient Initiative, Addis Ababa, Ethiopia; Micronutrient Initiative, Nairobi, Kenya

**Keywords:** ORS, Zinc, Bundling, Co-packing, Adherence, Ethiopia

## Abstract

**Background:**

Presumably bundling/co-packaging of zinc with ORS encourages the combined use of the products for diarrhea treatment; however, empirical evidences are scarce. The purpose of this work is to evaluate whether co-packing using a plastic pouch can enhance the joint adherence to the treatment or not. The study also compares the cost effectiveness (CE) of two co-packaging options: ‘central’ and ‘health center (HC)’ level bundling.

**Methods:**

This cluster-randomised controlled trial was conducted in 2015 in eight districts of Ethiopia. Thirty two HCs were randomly assigned to one of the following four intervention arms: *(i)* ‘Central bundling’ (zinc and ORS bundled using a pouch that had instructional message, distributed to HCs); *(ii)* ‘HC level bundling’ (zinc, ORS and a similar pouch distributed to the HCs and bundled by health workers); *(iii)* ‘Bundling without message’ (zinc, ORS and plain pouch distributed and bundled by the health workers); and, *(iv)* ‘Status quo’ (zinc and ORS co-administered without bundling). In each of the four arms, 176 children 6–59 months of age, presented with acute diarrhea were enrolled. Twelve days after enrollment, level of adherence was assessed. A composite scale of adherence was developed and modeled using mixed effects linear regression analysis. The unit costs associated with the arms were estimated using secondary data sources. Incremental CE analysis was made by taking the cost and level of adherence in fourth arm as a base value.

**Results:**

The follow-up rate was 95.6 %. As compared with the ‘status quo’ arm, the joint adherences in the ‘central’ and ‘HC level’ bundling arms raised substantially by 14.8 and 15.7 percentage points (PP), respectively (*P* < 0.05). No significant difference was observed between ‘bundling without message’ and the ‘status quo’ arms. The unit cost incurred by the ‘central bundling’ is relatively higher (USD 0.658/episode) as compared with the ‘HC level bundling’ approach (USD 0.608/episode). The incremental CE ratio in the ‘central bundling’ modality was two times higher than in the ‘HC based bundling’ approach.

**Conclusion:**

Bundling zinc with ORS using a pouch with instructional messages increases adherence to the treatment. ‘HC level bundling’ is more CE than the ‘central bundling’ approach.

**Electronic supplementary material:**

The online version of this article (doi:10.1186/s12889-016-3126-6) contains supplementary material, which is available to authorized users.

## Background

Diarrhea remains the second leading cause of death among infants and young children, accounting for 18 % of mortality and 13 % of all Disability-Adjusted Life Years [1]. Among children younger than 5 years, each year diarrhea kills around 760,000 children and causes 1.7 billion illnesses [2, 3]. Of all child deaths from diarrhea, 78 % occur in the Africa and South-East Asia [4].

Despite the recent decline in infant (50/1000 live births (LB)) and under five (88/1000 LB) mortality rates, in Ethiopia child morality still remains high [5]. In the country 22 % of childhood deaths are attributable to diarrhea [4]. Furthermore, according to Ethiopia Demographic Health Survey (DHS) 2011, 13 % children younger than 5 years had diarrhea in the preceding two weeks and the figure was as high as 25 % amongst children 6–11 months of age [5].

Despite the alarming statistics, diarrhea is highly treatable. More than three-quarters of all diarrhea deaths could be averted with full coverage and utilization of Oral Rehydration Salt (ORS) and adjunct zinc supplementation [6]. There is unequivocal evidence that therapeutic zinc supplementation decreases the duration and severity of diarrheal episode and the likelihood of subsequent infections in the 2–3 months following treatment [7–9]. Zinc supplementation also cut diarrhea related mortality by nearly one quarter [10].

Its recommend that under-five years old children should receive 10–14 days of zinc treatment for diarrhea [11]. However, an increasing number of studies are showing that adherence to zinc is unsatisfactory [12–16]. A study in Kenya reported that among mothers who received diarrhea treatment for their children, 62 % reported giving zinc for fewer than the recommended 10 days [12]. In Bangladesh two studies reported 56 % [14] and 62 % [15] adherence to the full ten days treatment course. In Mali, less than two-thirds (64 %) received the entire 14-day treatment [13]. In India, at the 7th day of treatment, 82 % of children had already stopped taking the supplement [16].

Bundling (co-packaging) of zinc with ORS may encourage their combined use, and enhance access to and utilization of the treatment [17, 18]. On-pack information can also serve as education and communication tool. A study conducted in Western Guatemala found, bundling of zinc with ORS in a graphic co-pack with instructions and provider messages for counseling significantly improved both the prescription practices of health professionals and adherence to zinc treatment of mothers [19]. Formative research in Cambodia also concluded that co-packaging is an effective means of encouraging the combined use zinc and ORS [20].

The gray literature indicates many countries in the developing world including Kenya, Uganda, Zambia, Nigeria, Benin, Cambodia and Guatemala are initiating co-packaging of zinc and ORS for diarrhea treatment. Likewise, in Ethiopia, with the support of Micronutrient Initiative (MI), the Pharmaceuticals Fund and Supply Agency (PFSA) has launched the distribution of the bundled products in selected regions of the country. Nevertheless, limited evidence exists regarding the effectiveness of the intervention.

Bundling of zinc and ORS can be implemented in two approaches. Readymade centrally bundled products can be distributed to the health institutions (Central bundling) or the products can be dispatched and health workers do the bundling at health institution level (health center (HC) level bundling). Nevertheless, comparative cost effectiveness (CE) has not been made.

Thus, the purpose of this study is to evaluate whether co-packing of zinc with ORS using a plastic pouch can enhance the joint adherence to diarrhea treatment or not. The study also compared the CE (cost per unit of a defined health outcome [21]) of ‘central’ and ‘health center (HC)’ level bundling approaches as a means to provide empirical evidence for decision making.

## Methods

### Trial design

The study was a cluster-randomised controlled trial with four parallel intervention arms and an allocation ratio of 1.

### Participants

The study was conducted in 32 HCs selected across 8 districts of Tigray, Amhara, Oromya and Southern Nations, Nationalities and Peoples (SNNP) regions of Ethiopia. In Ethiopia, HC is part of the primary health care system that provides preventive and basic curative services. The study districts were Laelay Maychew, Enda Mehoni, Tehuledere, Dera, Dendi, Dodola, Misrak Badwacho and Aleta Wondo.

Children 6–59 months of age presented with diarrhea in the HCs during the study period (January to March, 2015) where eligible for enrolment. Children presented with persistent diarrhea (lasting for 14 days or more), bloody diarrhea or severe dehydration [22] were excluded. Likewise, those who came outside the catchment area of the HCs or from inaccessible localities weren’t included.

### Intervention

**Fig. 1:**
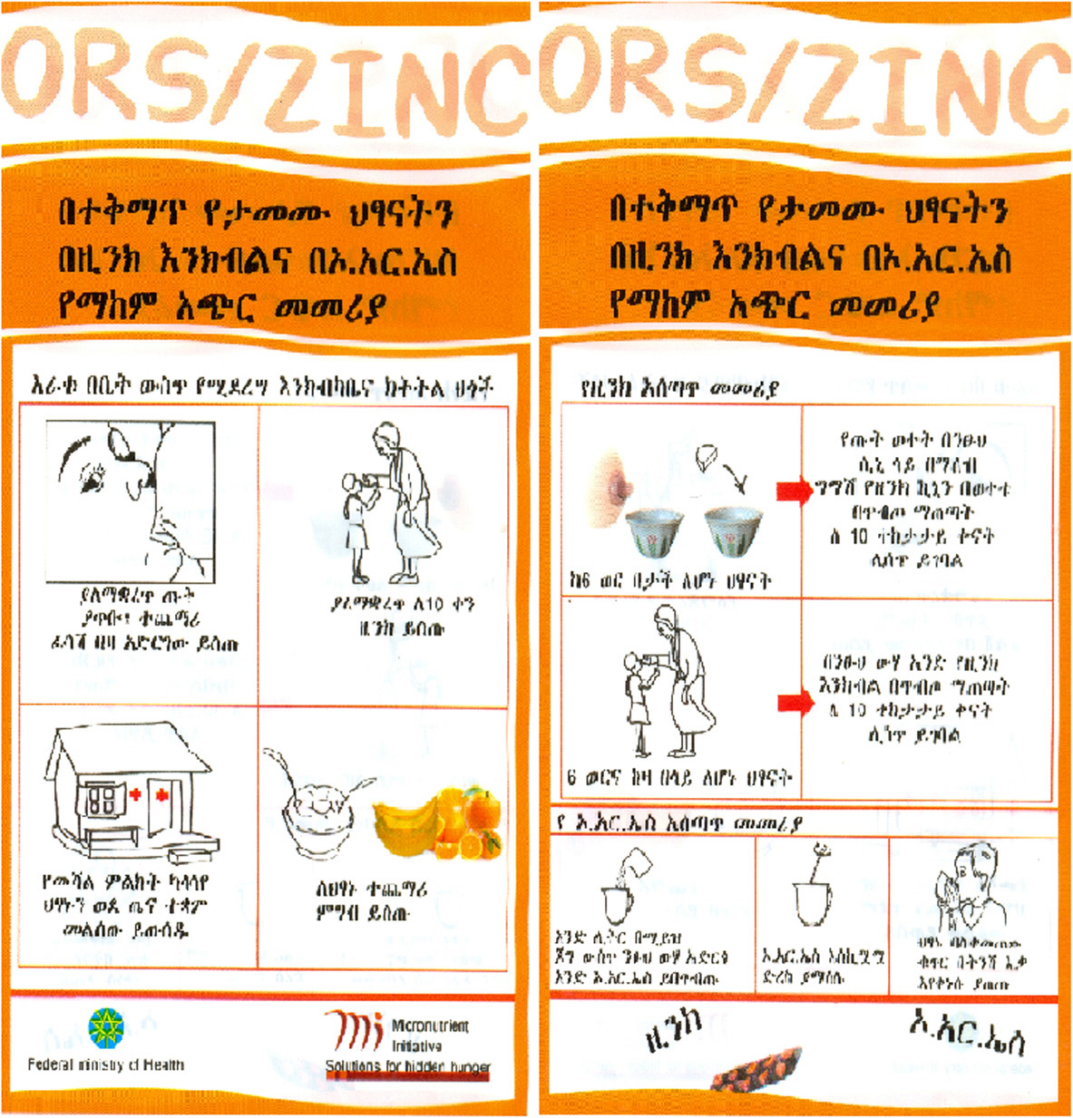
Design of the pouch used for bundling zinc with ORS

Thirty two HCs were randomly allocated to one of the following four parallel intervention arms:

1. Central bundling: Pre-bundled zinc and ORS using a pouch that had instructional message intended for improving the rational use of zinc-ORS treatment, distributed to HCs (Fig. 1).
2. HC level bundling: Zinc, ORS and bundling pouch distributed to the HCs and bundling was made by the health workers while administrating the treatment. The pouch also carries similar instructional messages as in the first arm.
3. Bundling without message: Zinc, ORS and plain pouches without messages distributed to the HCs and bundling was carried out by the health workers.
4. The status quo: Zinc and ORS co-administered without bundling.

Enrollment of cases was made on an ongoing basis while the caregivers visited the HCs for treatment. During first contact, baseline data were collected, treatment administered according to the study protocol and the address of the household was registered. Two weeks later, respondents were visited at their home and adherence was assessed based on the self report of parents/caregivers. Data were collected by 96 trained health professionals working in the selected HCs using a pretested questionnaire prepared in the local languages.

### Outcome of the study

The primary outcome of the study was level of joint adherence to zinc and ORS treatment; secondary outcomes were level of independent adherence to zinc and ORS treatment.

Adherence to zinc was calculated as percentage of zinc tablets given to the baby out of the ten provided at the HC; whereas, adherence to ORS was computed as percentage of times ORS was administered after the child had diarrheal episodes. Joint adherence to zinc and ORS measured using a composite index developed by assigning a weight of 0.5 to each of the zinc and ORS adherence index.

### Sample size

The sample size required for assessing the level of adherence and cost-effectiveness (CE) was computed via the GPower 3.15 software [23] using sample size calculation formula for comparison of means. The inputs were; 95 % confidence level, 90 % power, 0.3 effect size to be detected as significant, an allocation ratio of 1, design effect of 1.7 (calculated based on the expected intra-cluster correlation and cluster size [24, 25]) and 15 % compensation for loss to follow-up. Ultimately the sample size was computed as 704 children, i.e. 176 per each arm.

### Randomization

From each of the four regions (Tigray, Amhara, Oromya and SNNP) two districts were selected at random. Then from each district, 4 HCs having relatively high patient flow were selected. Then four of the HCs selected from each district were randomly allocated to the four arms via the lottery method. Randomisation was done by one of the principal investigators. Finally based on the eligibility criteria, 22 subjects were recruited from each HC using quota sampling technique.

### Blinding

The study did not involve any kind of blinding as it was not possible.

### Statistical methods

The data were entered and analyzed using SPSS 19.0 and STATA SE 12 software. The analysis was made according to the initial treatment arm assignment. Prior to comparing the level of adherence in the four arms, the socio-demographic profiles of the groups were compared. Categorical variables were evaluated using chi-square test; whereas numeric measures, depending on their normality, assessed using ANOVA or kruskal Wallis test.

The levels of adherence across the four intervention arms were compared using mixed effects multiple linear regression model (with random intercepts for region, district and HC) adjusted for two variables that showed significant variation across the arms. Prior to analysis the assumptions of linear regression were assessed.

Wealth index was computed using principal component analysis as a composite indicator of living standard based on ownership of selected household assets, size of agricultural land, quantity of livestock, materials used for housing construction, and ownership of improved water and sanitation facilities. Ultimately wealth tertiles were generated.

The unit costs associated with the three arms (excluding ‘bundling without message’ arm) were estimated based on different secondary data sources. Various direct costs (costs for purchasing zinc, ORS and bundling pouch; cost for printing the messages on the pouch) and indirect costs (costs of transportation for the aforementioned items, marginal manpower cost for conducting the bundling at central and HC levels, and intellectual cost for developing the message printed on the pouch) were estimated. The costs are calculated as an aggregate of all inputs which are identified, measured and thereafter valued at determined unit costs.

With the intension of making macro level comparisons, the hypothetical total annual expenses of the country for treating acute watery diarrhea using the three different approaches were estimated. The computation was made by multiplying the unit cost for treatment by the expected number of health institution based diarrhea treatments per annum. Information regarding the approaches used to estimate unit cost and total national cost is provided as an auxiliary file (Additional file 1).

Incremental CE analysis was made by taking the added Percentage Points (PP) of adherence for joint zinc-ORS treatment as the sole measure of effectiveness. The cost and level of adherence in the fourth arm (the usual way of distribution without bundling) was taken as the base value. CE ratio was calculated by dividing the added unit costs with the added PP in adherence.

## Results

### Characteristics of the respondents and the study subjects

In the baseline study 704 children (176 from each arm) were enrolled. In vast majority of the cases, data were collected from the mothers of the index children. The sex and age profiles of the children were not significantly different (*P > 0.05*). However, with the average male to female ratio of 1.22, more boys than girls were enrolled. The variation in the mean (±SD) age of the respondents was marginally insignificant (*p = 0.063*). Proportion who fell in the poor household wealth tertile ranged from 30.1 % in the ‘status quo’ to 36.4 % in the ‘HC level bundling’ group (*p = 0.097*). The distribution of socio-demographic characteristics of the cases and respondents during the baseline survey was not significantly different (Table 1).

**Table 1:** Characteristics of the respondents and the cases during the baseline survey in the four arms of the study, Jan-Mar 2015

Variables	Arms				*P* value
	Central bundling (*n* = 176)	HC level bundling (*n* = 176)	Status quo (*n* = 176)	Bundling without message (*n* = 176)	
Type of the respondent (%)					
Mothers	92.0	89.8	92.0	92.0	0.832
Other primary caregiver	8.0	10.2	8.0	8.0	
Mean age (± SD) of the respondents (years)	28.2 (±7.6)	26.9 (±6.3)	28.4 (±6.7)	26.8 (±6.9)	0.063
Mean parity (± SD)	3.4 (±2.1)	3.0 (±2.0)	3.4 (±2.1)	3.3 (±2.1)	0.266
Educational status (%)					
No formal education	51.1	46.6	51.7	53.4	0.231
Primary education	27.8	27.3	21.6	29.5	
Secondary education or above	21.0	26.1	26.7	17.0	
Occupation (%)					
Housewife/farmer	90.0	93.2	86.4	90.3	0.187
Others	9.1	6.8	13.6	9.7	
Marital status (%)					
Married/living together	92.6	92.6	96.0	95.5	0.364
Others	7.4	7.4	4.0	4.5	
Household wealth index (%)					
Poor	31.8	36.4	30.1	34.7	0.097
Middle	28.9	34.7	40.9	29.0	
Rich	39.3	29.0	29.0	36.4	
Age of the child (months) (%)					
6–11	39.3	30.7	30.1	34.1	0.151
12–23	39.3	42.6	39.8	46.6	
24–59	21.4	26.7	30.1	19.3	
Sex of the child (%)					
Boys	59.0	55.1	53.4	52.3	0.614
Girls	41.0	44.9	46.6	47.7	

**Fig. 2:**
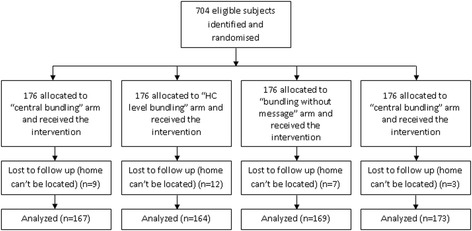
Flow diagram of the study

About 673 (95.6 %) of the cases were successfully followed and household data were collected. The remaining cases could not be traced during the household interviews. The follow-up rates ranged from 93.2 % in the ‘HC level bundling’ to 98.3 % in ‘bundling without message’ arms, but the figures were not significantly different (*p = 0.123*). The median duration between the baseline and follow-up interviews was 12 days and varied from 10 to 23 days (*p = 0.163*). The socio-demographic characteristics of the subjects who completed the follow-up were not statistically different (Fig. 2).

### Severity and duration of the diarrheal illness

Severity of illness and other treatments provided might independently affect the adherence to zinc-ORS treatment. In overall, the arms were significantly different based on the number of reported episodes of diarrhea in the preceding day of the baseline survey (*p < 0.001*) and the extent of dehydration during initial presentation (*p = 0.004*). Differences on the median duration of illness at the time of the baseline study were marginally insignificant (*p = 0.069*). During enrollment, children from the ‘HC level bundling’ arm appear to have a longer and more frequent diarrheal episodes and a higher proportion exhibited signs of dehydration (Table 2).

**Table 2:** Severity and duration of diarrhea among study children across the four intervention arms during the baseline survey, Jan-Mar 2015

Variables	Arms				*P* value
	Central bundling (*n* = 167)	HC level bundling (*n* = 164)	Status quo (*n* = 169)	Bundling without message (*n* = 173)	
Median duration of illness during the baseline study (days)	3	3	2	2	0.069
Mean (±SD) number of episodes in the preceding day of survey	5.3 (±1.4)	6.2 (±2.5)	6.0 (±2.5)	5.5 (±2.0)	<0.001*
Extent of dehydration (%)					
No dehydration	74.9	65.2	76.9	82.1	0.004*
Some dehydration	25.1	34.8	23.1	17.9	
% who sought for treatment elsewhere before coming to HC	4.8	9.1	4.7	8.1	0.247
% who received antibiotic or antiparasitic drugs at the HC	35.9	39.0	44.4	39.3	0.461

* Statistically significant difference at *p* value of 0.05

Despite the assumption that children with acute watery diarrhea typically don’t need antibiotic and antiparasitic medications, very significant proportion (ranging from 35.9 to 44.4 %) received such treatments. Nevertheless, the figures were not statistically different across the arms (*p = 0.257*). The proportion of caretakers who sought treatment elsewhere prior to coming to the study HCs were not significantly different (*p = 0.247*).

### Adherence to zinc supplementation

The caretakers were asked to quantify how many of the 10 zinc tablets prescribed were actually given to the child. In the ‘status quo’ arm only 37.9 % of the children received the full ten days treatment course. The corresponding figures for the ‘bundling without message’, ‘HC level bundling’ and ‘central bundling’ were 45.7 %, 64.6 % and 65.9 %, respectively.

**Fig. 3:**
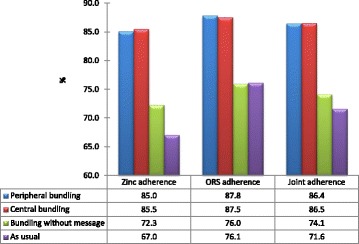
Independent and joint adherence to zinc and ORS across the four intervention arms of the study, Jan-Mar 2015

On average, across the four arms, the average (±SD) number of tablets given was reported as 7.70 (±2.90). Based on ANOVA, the adherence level was significantly higher in the ‘HC level bundling’ (8.55 ± 2.31) and ‘central bundling’ (8.50 ± 2.56) groups; and lower in ‘bundling without message’ (7.23 ± 3.19) and ‘status quo’ (6.70 ± 3.36) arms (*p < 0.001*). No difference was observed between the latter two arms (*p = 0.557*). Likewise, the variation between the ‘central’ and ‘HC level’ bundling groups was insignificant (*p = 0.999*) (Fig. 3).

The level of zinc adherence in the intervention arms was also compared using a mixed effects multivariate linear regression model in which adjustments were made for two potential confounders (number of episodes of diarrhea and extent of dehydration during the first presentation). Compared with the ‘bundling without message’ group, ‘central bundling’ and ‘HC level bundling’ significantly increased zinc adherence by 1.35 and 1.41 tablets (i.e. by 13.5 and 14.1 PP, respectively), respectively. Likewise, compare with the ‘status quo’ group, the two groups significantly increased adherence by 1.76 and 1.94 tablets (i.e. by 17.6 and 19.4 PP), respectively. No difference was observed between the ‘status quo’ and ‘bundling without message’ arms (*p = 0.319*); and between ‘central bundling’ and ‘HC level bundling’ arms (*p = 0.894*) (Table 3).

**Table 3:** Pairwise comparison of the intervention arms using mixed effects multivariate linear regression model based on zinc, ORS and joint adherence, Jan-Mar 2015

Pairwise combination	*P* value	*β* coefficient ^a^
Zinc adherence (%)		
Central bundling vs HC level bundling	0.894	−0.4
Central bundling vs Bundling without message	0.001^*^	13.5
Central bundling vs Status quo	<0.001^*^	17.6
HC level bundling vs Bundling without message	0.002^*^	14.1
HC level bundling vs Status quo	<0.001^*^	19.4
Bundling without message vs Status quo	0.319	4.2
ORS adherence (%)		
Central bundling vs HC level bundling	0.969	0.2
Central bundling vs Bundling without message	0.007^*^	11.5
Central bundling vs Status quo	<0.001^*^	12.0
HC level bundling vs Bundling without message	0.022^*^	10.2
HC level bundling vs Status quo	0.003^*^	12.1
Bundling without message vs Status quo	0.966	−0.3
Joint zinc and ORS adherence (%)		
Central bundling vs HC level bundling	0.965	−0.1
Central bundling vs Bundling without message	0.002^*^	12.6
Central bundling vs Status quo	<0.001^*^	14.8
HC level bundling vs Bundling without message	<0.001^*^	12.0
HC level bundling vs Status quo	<0.001^*^	15.7
Bundling without message vs Status quo	0.673	1.8

* Statistically significant association at *p* value of 0.05

^a^ Unstandardized multiple linear regression coefficient adjusted for number of episodes of diarrhea and level of severity of dehydration

### Adherence to ORS

According to the national guideline for the treatment of acute watery diarrhea, children should receive ORS after every diarrheal episode. About 63.9 % of the respondents from the ‘status quo’ arm reported that they provided ORS every time after an episode as recommended. The corresponding figures for the ‘bundling without message’, ‘central bundling’ and ‘HC level bundling’ were 64.2, 79.0 and 80.5 %, respectively.

Overall, the average adherence levels for ORS treatment in the four arms (i.e. proportion of diarrheal episodes followed by ORS administration) were: ‘HC level bundling’ (87.8 %), ‘central bundling’ (87.5 %), ‘status quo’ (76.1 %) and ‘bundling without message’ (76.0 %).

In the mixed effects multivariate linear regression model adjusted for the two potential confounders, no significant difference was observed on the level of ORS adherence between ‘central’ and ‘HC level’ bundling (*p = 0.969*) and ‘bundling without message’ and ‘status quo’ interventions groups (*p = 0.966*). However, as compared with the other two groups, the ‘central’ and HC level’ bundling interventions increased ORS adherence by 10.2 to 12.0 PP (*P* < 0.05).

### Adherence to joint zinc-ORS treatment

The intent of co-packing is to enhance the combined use zinc and ORS for diarrhea treatment and to avoid the use of zinc as a replacement therapy for ORS. Hence assessing the joint adherence is imperative. The joint adherence level in the ‘central’ (86.4 %) and ‘HC level’ bundling (86.5 %) arms was comparable and significantly higher than the level in the other two arms. The joint adherence in the ‘bundling without message’ (74.1 %) appears to be higher than the corresponding figure (71.6 %) in the ‘status quo’ arm, but the difference was not statistically significant.

In the mixed effects multivariate linear regression model, similar pattern of association was witnessed. As compared with the ‘status quo’ arm, the joint adherences to zinc and ORS in ‘central’ and ‘HC level’ intervention groups were significantly increased by 14.8 and 15.7 PP, respectively. Likewise, taking the ‘bundling without message’ as the reference group, the joint adherence level was increased by 12.6 and 12.0 PP both in ‘central’ and ‘HC level’ arms.

### Reasons for not adhering to zinc-ORS treatment

Among 300 caregivers who missed 2 or more zinc tablets the underlying reasons were appraised. The most frequently mentioned causes were; recovery of the child (42.3 %), the child did not like it (25.3 %), forgetfulness (21.7 %), and occurrence of perceived side effects (13.0 %). Similarly among 190 respondents who at least once failed to give ORS after a diarrheal episode, the underlying reasons for not doing so were; the child did not like it (52.6 %), thinking that ORS does not help the child much (20.0 %), forgetfulness (12.6 %), underestimating the seriousness of the disease (10.5 %) and giving preference to homemade fluids (7.9 %).

### Estimated unit and total national cost for diarrhea treatment

The costs associated with the three strategies (central bundling, HC level bundling and the status quo) were estimated based on various assumptions. The unit cost incurred by the ‘central bundling’ approach is relatively higher (USD 0.658/episode) as compared with that of ‘HC level bundling’ approach (USD 0.608/episode). Expectedly, the ‘status quo’ modality is cheaper (USD 0.556/episode).

In terms of the total national cost, the ‘status quo’ approach requires the health sector to invest USD 6,283,474 for diarrhea treatment per annum, given all the assumptions made for estimation are kept constant. The ‘HC level bundling’ and the ‘central bundling’ require an additional USD 559,256 and 1,156,184 investments, respectively. Likewise, the ‘central bundling’ as compared to the ‘HC level bundling’ approach requires an additional USD 596,928 per annum.

### Incremental CE of the central and HC level approaches

The incremental CE of the ‘central’ and ‘HC level’ bundling approaches was computed by taking the ‘status-quo’ as the base level. CE ratio was calculated by dividing added unit costs with the associated PP improvement in joint zinc-ORS adherence. The ratios for the ‘central’ and ‘HC level’ bundling approaches were 0.007 and 0.003, respectively. These can be interpreted as, in central and HC level bundling approaches, a PP improvement in joint Zinc-ORS adherence can be achieved by investing 0.7 and 0.3 cents/diarrhea episode, respectively. The Cost-effectiveness ratio was two times higher in the ‘central bundling’ modality as compared with the counterpart. The evidence shows, ‘HC level bundling’ is more cost-effective than ‘central bundling’ (Table 4).

**Table 4:** Comparison of CE ratios in central and HC level bundling approaches

Intervention approaches	Unit cost of treatment (USD/episode)	Joint adherence to zinc-ORS (%)	Increased unit cost (USD/episode)	Increased adherence (%)	CE ratio
Status quo	0.5562	71.6	– ^a^	– ^a^	– ^a^
HC level bundling	0.6057	86.5	0.0495	14.9	0.0033
Central bundling	0.6585	86.4	0.1023	14.8	0.0069

^a^ Set as the base value

## Discussion

Co-packaging of zinc with ORS can enhance adherence to treatment in a couple of ways. The bundling can create a notion that zinc and ORS are parts of a protocol and should not be used as stand-alone products; further, on-pack instructions can serve as an information and education tool. In the current study, the better adherence observed in the ‘central’ and ‘HC level’ arms and the absence of sizable difference between the other two arms demonstrate that the benefit is predominantly due to the effect of instructional messages provided on the pouch.

The positive effect of bundling zinc with ORS using a pouch with instructional messages has also been witnessed elsewhere. A study in Guatemala reported that provision of the products in a graphic co-pack with instructions significantly improved both the prescription practices and adherence to zinc. Mothers who received the co-pack had 1.7 times increased probability of providing the full 10 days of zinc treatment than their counterparts [19]. Experiences of social marketing of zinc in Cambodia also showed that co-packaging and an instructional insert had encouraged the combined use of the products [20].

In this study the level of zinc, ORS and joint adherences were comparable in the central and HC level bundling arms. However, the CE was better in the latter. Nevertheless, from program implementation point of view, apart from CE, other feasibility parameters including sustainability and acceptance of the task by health workers and PFSA should be evaluated.

As the study followed an interventional design, the reported level of adherence is unlikely to illustrate the existing practice in the study districts. Nevertheless, the ‘status quo’ arm may provide surrogate information. In this arm, merely 38 % of the respondents adhered to the full ten days zinc treatment indicating compliance is suboptimal. Increasing numbers of studies are witnessing the same. For instance, the complete adherence to the full 10 days zinc treatment course was as low as 29 % in Madagascar [26], 38 % in Kenya [12] and 44 % in Bangladesh [15]. Conversely, better level of adherence had been reported in Mali [13] and Nigeria [27].

In the current study the most frequently forwarded reason for discontinuation of zinc is recovery of the child. The fact that diarrheal illness typically lasts for few days but zinc is expected to be provided for 10–14 days creates a challenge for adherence. Another study based on a synthesis of formative research results from nine countries including Ethiopia also concluded that, the major barriers to the full course of zinc include the common practice of stopping treatment when diarrhea stops and a lack of caregiver awareness of the benefits of zinc for prevention of diarrhea [28]. It is therefore important to include in promotional material, strong communication on the need to complete the dosage regardless of whether the child’s diarrhea has ceased.

The findings of the study should be interpreted in consideration of its limitations. Adherence was assessed based on the caregivers’ report. Consequently, the level is likely to be overestimated due to social desirability bias. Further, adherence to ORS was assessed based on the proportion of diarrhea episodes that were followed by ORS administration. Remembering the total number of diarrheal episodes is evidently challenging and its liable recall errors. The data were only collected from HCs hence the findings may not be inferred to other types of health institutions as the type of health provider and clients’ characteristics may vary.

Regarding the CE analysis, the following limitations should be noted. The analysis is made by taking the number of added units of zinc-ORS adherence as the sole measure of effectiveness. Hence, any contribution of the intervention in terms of reducing the severity and recurrence of diarrhea has not been considered. Further, the estimated cost cannot be inferred to other settings as some of its components (like transportation cost) were peculiar to the study districts.

## Conclusion

Bundling zinc with ORS via a pouch with instructional messages increases adherence to diarrhea treatment. ‘HC level bundling’ is more cost effective than the ‘central bundling’ strategy. Bundling is a feasible approach for enhancing adherence to diarrhea treatment.

## Abbreviations

ANOVA, analysis of variance; CE, cost effectiveness; DHS, Demographic Health Survey; HC, health center; IRBs, Institutional Review Boards; LB, live births; MI, micronutrient initiative; ORS, Oral Rehydration Salt; PFSA, Pharmaceuticals Fund and Supply Agency; PP, percentage points; SD, standard deviation; SNNP, Southern Nations, Nationalities and Peoples; USD, United States Dollar

## Additional files

Additional file 1: Description of cost assumption and estimation. (DOCX 17 kb) Additional file 2: The dataset supporting the conclusions of the article. (XLS 481 kb) Additional file 3: The code book for the dataset supporting the conclusions of the article. (XLSX 12 kb)
