# Supplementary material for: The effectiveness bundling of zinc with Oral Rehydration Salts (ORS) for improving adherence to acute watery diarrhea treatment in Ethiopia: cluster randomised controlled trial
Source: BMC Public Health. 2016 May 31;16:457. doi: 10.1186/s12889-016-3126-6 (PMC4888310; doi:10.1186/s12889-016-3126-6)
Supplement: Additional file 1: — Description of cost assumption and estimation. (DOCX 17 kb) [file 12889_2016_3126_MOESM1_ESM.docx]

**Cost assumptions and estimation**

Estimation of cost associated with the three arms is made based on the following assumptions.

1. Cost for purchasing the bundling pouch: The price was computed based on the secondary data acquired from Micronutrient Initiative (MI) Ethiopia.
2. Personnel cost for bundling: The personnel cost for the ‘peripheral bundling’ approach was estimated by assuming that a health worker takes 5 seconds to complete bundling for a diarrhea treatment. The estimate was made based on field observations. Later, the time was changed into monitory values based on the existing overtime payment rate of the Ethiopian government. The personnel cost for the ‘central bundling’ approach was estimated based on the financial support provided by MI to Pharmaceuticals Fund and Supply Agency (PFSA) to cover the personnel cost for central bundling.
3. Cost for transportation of zinc, ORS and bundling pouch: The cost was estimated based on: (i) the weights of 1 zinc strip, 2 sachets of ORS, 1 plastic pouch; (2) gasoline needed for transportation (3) unit price of gasoline, (4) estimated distance for distributing the supply to the study districts, and (5) per diem for drivers.
4. Price of zinc and ORS: The unit cost was determined based on review of literatures. In this study, the price estimated by Clinton Health Access Initiative (10.25 birr per 2 sachets of ORS and 1 strip of zinc) [1] was used as its recent and compatible with other global estimates [2], [3].
5. Cost of orientation training for initiating the bundling program: The estimation was on the assumption that annually there would be an orientation/training of personnel for initiating and sustaining the bundling program. The orientation is likely to be integrated into the existing trainings; hence, only incremental costs are considered. For the ‘central bundling’ approach, annually 30 personnel (10 from the central PFSA hub and 20 from the other 10 branch hubs) at least need to be oriented for a day and they will receive 210 ETB/person as per dime. For the ‘peripheral bundling’ approach; from the existing 70 zones of the country 1 health professional and from the existing 3,285 health centers 1 health worker will receive an annual half a day orientation and will receive 105 br/person per diem. For both approaches 10% contingency was added to accommodate other expenses associated with the training. The total training cost was converted into unit price by dividing it by the expected number of diarrhea cases in the country per annum.
6. Intellectual cost: The intellectual cost for developing and validating the print message was also considered in the cost estimation. The total cost spent by MI for the task was converted into unit cost by dividing it with the total number of diarrhea cases expected in the country over 5 years period. This is made based on the assumption that the BCI message will be revised every 5 years.

**Estimation of the total national cost for treating diarrhea using the three approaches**

For the intension of making macro level comparisons, the total annual expenses of the country for treating acute watery diarrhea using the three different approaches (central bundling, peripheral bundling and the status quo) were estimated. The computation was made by multiplying the unit cost for treatment by the expected number of health institution based diarrhea treatments per annum. The estimation of the expected number of diarrhea treatments was made based on the following assumptions:

1. According to a study conducted in 2012 [4], in Sub-Saharan Africa 3 episodes of diarrhea occur per child/day. The figure was also adopted to Ethiopia;
2. Nevertheless, after the aforementioned study, Rota vaccine had been introduced into the Ethiopia’s Expanded Program for Immunization (EPI). This would intuitively reduce the incidence of diarrhea in the country. Accordingly the incidence (3 episodes/per child/day) was corrected for the expected coverage and preventive efficacy of the vaccine. The Rota vaccine coverage in 2015 was assumed to be 56.2%. The figure was estimated by projecting the DPT-2 coverage reported in DHS 2005 [5] and 2011 [6] surveys to the year 2015 as both vaccines are administered at the same schedule. It was also assumed that the vaccine reduces the incidence of diarrhea by 42% [7]. Ultimately the corrected incidence of diarrhea per child/year for the country was 2.5;
3. As of 2015 the country’s population is 95.9 million [8] and under five children contribute to 15% of the population [9];
4. Based on the health care seeking behavior for diarrhea treatment, about 31.0% of the episodes are expected to be treated by ORS and zinc. The figure was projected to the year 2015 based on the data of DHS 2000 [10] and DHS 2011 [6].

**References**

1. Clinton Health Access Initiative. Program areas: zinc and ORS, January 2015. Available from: <http://www.clintonhealthaccess.org/program-areas/maternal-and-child-health/zinc-ors>. Date accessed: May 01, 2015.
2. Zinc and health blog. February 2015. Available from <http://www.zincsaveslives.com/Generic.aspx?PAGE=Blog&portalName=zinc>. Date accessed: May 01, 2015.
3. ENN. Field exchange: Scaling up ORS and zinc treatment for diarrhea reduces mortality. December 2012. Available from <http://www.ennonline.net/fex/44/scaling>. Date accessed: March 03, 2015.
4. Walker CL, Perin J, Aryee MJ, Boschi-Pinto C, Black RE. Diarrhea incidence in low- and middle-income countries in 1990 and 2010: A systematic review. BMC Public Health. 2012; 12:220.
5. ORC Macro, Central Statistical Agency [Ethiopia]. Ethiopia demographic and health survey 2005. Addis Ababa and Calverton: Central Statistical Agency and ORC Macro; 2006.
6. Central Statistical Agency [Ethiopia] and ICF International. Ethiopia demographic and health survey 2011. Addis Ababa, Ethiopia and Calverton, USA; 2012.
7. Soares-Weiser K, MacLehose H, Bergman H, Ben-Aharon I, Nagpal S, Goldberg E, *et al*. Vaccines for preventing rotavirus diarrhoea: vaccines in use. Cochrane Database Syst Rev. 2012; 11: DOI: 10.1002/14651858.CD008521.pub3.
8. Population Reference Bureau. World population data sheet, 2014. Available from: [http://www.who.int/water_sanitation_health/publications /factsfigures04/en/](http://www.who.int/water_sanitation_health/publications%20/factsfigures04/en/). Data accessed: Jan 17, 2015.
9. Federal Republic of Ethiopia Census Commission. Summary and statistical report of the 2007 population and housing census: Population size by age and sex. Addis Ababa; 2008
10. ORC Macro, Central Statistical Agency [Ethiopia]. Ethiopia demographic and health survey 2000. Addis Ababa and Calverton: Central Statistical Agency and ORC Macro; 2001.
